# Comparative Transcriptomics of *H. pylori* Strains AM5, SS1 and Their *hpyAVIBM* Deletion Mutants: Possible Roles of Cytosine Methylation

**DOI:** 10.1371/journal.pone.0042303

**Published:** 2012-08-03

**Authors:** Ritesh Kumar, Asish K. Mukhopadhyay, Prachetash Ghosh, Desirazu N. Rao

**Affiliations:** 1 Department of Biochemistry, Indian Institute of Science, Bangalore, India; 2 Division of Bacteriology, National Institute of Cholera and Enteric Disease, Kolkata, India; New England Biolabs, Inc., United States of America

## Abstract

*Helicobacter pylori* is an important human pathogen and one of the most successful chronic colonizers of the human body. *H. pylori* uses diverse mechanisms to modulate its interaction with the host in order to promote chronic infection and overcome host immune response. Restriction-modification genes are a major part of strain-specific genes present in *H. pylori*. The role of N^6^ - adenine methylation in bacterial gene regulation and virulence is well established but not much is known about the effect of C^5^ -cytosine methylation on gene expression in prokaryotes. In this study, it was observed by microarray analysis and RT-PCR, that deletion of an orphan C^5^ -cytosine methyltransferase, *hpyAVIBM* in *H. pylori* strains AM5and SS1 has a significant effect on the expression of number of genes belonging to motility, adhesion and virulence. AM5Δ*hpyAVIBM* mutant strain has a different LPS profile and is able to induce high IL-8 production compared to wild-type. *hpyAVIBM* from strain 26695 is able to complement mutant SS1 and AM5 strains. This study highlights a possible significance of cytosine methylation in the physiology of *H. pylori*.

## Introduction

*Helicobacter pylori* is known to be involved in chronic gastritis, peptic ulcer diseases and in the multi-step carcinogenic process of gastric cancer [1]. Around 50% of the world population carries *H. pylori* and develops persistent inflammation in their stomachs, which lasts for decades unless treated with antibiotics [2]. Although almost all *H. pylori* infected individuals develop gastritis [3], it is still an enigma why few strains are associated with ulcer formation with relevant clinical symptoms, while majority of the *H. pylori* infected individuals remain asymptomatic. *H. pylori* is a genetically diverse species due to its natural competence and high mutation and recombination frequencies [4]. A large number of genes encoding restriction-modification (R-M) systems are found in the genome of *H. pylori*. R-M genes comprise approximately 10% of the strain-specific genes, but the relevance of having such an abundance of these genes is not clear [5].

DNA methylation is one of the most significant modifications of DNA bases [6]. The methylation pattern plays a significant role in controlling the gene expression [7]. Methylation of adenine alters the DNA curvature and decreases the stability of the DNA. This change in DNA conformation and structure in turn affects the interaction between proteins and DNA, especially for those DNA interacting proteins for which DNA sequence and structure is necessary [8]. In case of the prokaryotes, it is DNA adenine methylation that has been shown to affect the interaction between DNA and DNA binding proteins like RNA polymerases and transcription factors [7]–[8]. The roles of CcrM (cell-cycle regulated methyltransferase) in cell-cycle regulation and Dam (DNA adenine methyltransferase) in DNA repair, replication and gene regulation are well established [8]–. Other than N^6^ methyl adenine, C^5^ methyl cytosine and N^4^ methyl cytosine are two other methylated bases commonly found in prokaryotic genome [6]. Compared to N^6^ methyl adenine, the role of these two methylated bases in gene regulation is less known. In contrast to prokaryotes, C^5^ methyl cytosine is very important in epigenetic regulation in eukaryotes [11]. It has been shown that in some bacteria, cytosine MTase (Dcm) is associated with very short patch repair (Vsr) [12].

*H. pylori* genome has a number of R-M systems. *H. pylori* is well adapted to the gastric environment, and acquisition of numerous R-M systems might be related to its unique lifestyle [13]–[14]. Most of the DNA methyltransferases present in *H. pylori* are N^6^ adenine methyltransferases. A number of reports have shown that N^6^ adenine methyltransferases are important in the physiology of *H. pylori* and have a role beyond genome protection [15]–[16]. It has been shown that levels of *hpyIM* expression vary with the growth phase with higher expression during exponential growth than during stationary phase. Inactivation of *hpyIM* results in pleiotropic bacterial morphology including alteration in the expression of the *H. pylori dnaK* stress-responsive operon [15]. Comparison between *hpyAIVM* mutant and wild-type strains has revealed two genes, *katA* (HP0875) and HU (HP0835) to be down-regulated in the mutant strain [16]. Deletion of *hpyAVIAM*, an N^6^ adenine MTase in strain 26695 results in a slow growth phenotype, suggesting a possible role of this MTase in gene regulation [17].

In *H. pylori* strain 26695, *hpyAVIBM* is a C^5^ cytosine methyltransferase that exists as an overlapping ORF with another methyltransferase *hpyAVIAM* [13]. These MTases are believed to be remnant MTases of a defunct R-M system [18]–[19]. Both these ORFs have a high similarity with MnlI DNA MTase, which belongs to Type IIS R-M system [18]. However, in *H. pylori* the functional MnlI restriction enzyme homolog is absent [19]. *hpyAVIBM* has a stretch of dinucleotide repeats (AG), which makes it a candidate for phase variation [20]. Phase variation plays a vital role in a number of pathogenic bacteria, as it is used to facilitate immune evasion in a host and environmental adaptation [21].

These considerations and our special interest regarding the possible involvement of phase variable R-M systems in *H.pylori* pathogenesis motivated the present study to examine the role of cytosine methylation by HpyAVIBM MTase in two *H. pylori* strains, AM5 and SS1. This study highlights the significance of cytosine methylation in gene regulation and emphasizes that DNA methylation could be playing an important role in gene regulation in a pathogen like *H. pylori* that has a small genome with few regulatory proteins and small RNA [5], [22].

## Materials and Methods

### Bacterial Strains and Growth Conditions

*H. pylori* cultures were grown on petri plates containing brain heart infusion (BHI) agar (Difco) with horse serum (Invitrogen), isovitalex, and antibiotics (Vancomycin (6 µg/ml, Trimethoprim (8 µg/ml, and Polymyxine B sulphate 2.5 U/ml) and transformation by electroporation was done as explained earlier [23]. For motility studies, BHI broth containing 0.35% agar was used and experiment was done as explained earlier [23]. Motility assay was done in duplicates with three independent biological replicates.

### DNA Manipulation and Analysis

Chromosomal DNA from bacterial pellets was prepared from confluent growth on BHI agar plate cultures by the cetyltrimethylammonium bromide extraction method [24]. PCR for detection of the *hpyAVIBM* allele was carried out by using the appropriate primers (primers 1 to 4, Table S1). Positive and negative controls were included in each assay. PCR products were sequenced. Rapid Amplified Polymorphic DNA (RAPD) analysis was done by using primers 35–38 (Table S1).

### Construction of a Δ*hpyAVIBM* Mutant Strain

The 1064 bp long *hpyAVIBM* gene was amplified from genomic DNA of *H. pylori* 26695,AM5 and SS1 strains by polymerase chain reaction with Pfu polymerase using primer 1 and 2 (Table S1). The primers were designed with the help of the annotated complete genome sequence of *H. pylori* 26695, considering the putative gene sequence of *hpyAVIBM*, obtained from TIGR [25]. The amplified PCR fragment was ligated into the SmaI site of pUC19 and then inserted into the bacterial expression vector pET28a at the BamHI and XhoI sites. pET28a-*hpyAVIBM* plasmid was digested with AvrII and PstI, to release a fragment of 50 bp from *hpyAVIBM* leaving an overhang of 290 bp and 728 bp at both ends with pET28a vector backbone. The chloramphenicol cassette was obtained from plasmid DR2 (PCR amplified chloramphenicol cassette from pHel2 was ligated into the SmaI site of pUC19 to get DR2 plasmid) by using enzymes XbaI and PstI, and ligated with digested pET28a-*hpyAVIBM* plasmid. *hpyAVIBM::cat* construct was amplified from pET28a-*hpyAVIBM*::*cat* plasmid by using primers 1 and 2 (Table S1) and this was used for electroporation as described earlier [23]. Specific PCR for scoring of mutant alleles was carried out using appropriate primers (primers 1 and 2, primers 5 and 6; Table S1).

### Microarray Analysis

Bacterial RNA was stabilized *in vivo*, by using RNA protect Bacteria Reagent (Qiagen). Total RNA was isolated by using RNeasy Kits for RNA purification (Qiagen) as per the manufacturer’s protocol. Total RNA integrity was assessed using RNA 6000 Nano Lab Chip on the 2100 Bioanalyzer (Agilent, Palo Alto, CA) following the manufacturer’s protocol. Total RNA purity was assessed by the NanoDrop® ND-1000 UV-Vis Spectrophotometer (Nanodrop technologies, Rockland, USA). Total RNA with OD260/OD280>1.8 and OD260/OD230≥1.3 was used for microarray experiments. RNA samples with the rRNA 23S/16S ratios greater than or equal to 1.5 and with an RNA integrity number (RIN) higher than 7 were taken and Poly (A)-tails were added to the 3¢-end of RNA by using A-plus Poly (A) polymerase tailing kit (Epicentre Biotechnologies). Then the samples were labeled using Agilent Quick Amp Kit PLUS (Part number: 5190–0442). Five hundred nanograms each of the samples were incubated with reverse trancription mix at 42°C and converted to double stranded cDNA primed by oligodT with a T7 polymerase promoter. The cleaned up double stranded cDNA was used as template for cRNA generation. cRNA was generated by *in vitro* transcription and the dye Cy3 CTP (Agilent) was incorporated during this step. The cDNA synthesis and *in vitro* transcription steps were carried out at 40°C. Labeled cRNA was cleaned up and quality assessed for yields and specific activity. The labeled cRNA samples were hybridized onto a Custom Gene Expression *H. pylori* 8x15k (AMADID: **22857**). Six hundred ng of cy3 labeled samples were fragmented and hybridized. Fragmentation of labeled cRNA and hybridization were done using the Gene Expression Hybridization kit from Agilent (Part Number 5188–5242). Hybridization was carried out in Agilent’s Surehyb Chambers at 65°C for 16 hours. The hybridized slides were washed using Agilent Gene Expression wash buffers (Part No: 5188–5327) and scanned using the Agilent Microarray Scanner G Model G2565BA at 5 micron resolution. Data extraction from Images was done using Feature Extraction software v 10.5.1 of Agilent.

### Microarray Data Analysis

Feature extracted data was analyzed using GeneSpring GX v 10.0.2 software from Agilent. Normalization of the data was done in GeneSpring GX using the percentile shift and Normalize to Specific Samples. Genes that were significantly up and down regulated among the samples were identified. Differentially regulated genes were clustered using hierarchical clustering to identify significant gene expression patterns. Microarray experiments were done with biological replicates of both strains and their respective deletion mutants. Complete data has been submitted to GEO and the assigned accession number is GSE27946. All data is MIAME compliant.

### Semi Quantitative RT PCR

Reverse transcription (RT) was performed on 2 µg of total RNA by using the RevertAid™ H Minus First Strand cDNA synthesis kit (Fermentas) as per the manufacturer’s protocol. Of the cDNA, 2 µl was used in separate PCR reactions of 20 µl for each gene. To exclude the presence of DNA, for each sample the complete RT-PCR procedure was also carried out without adding reverse transcriptase. Data presented is the average of three biological replicates. Primer sequences are provided in supplementary section (Table S1). Densitometry was performed using the ImageJ gel analysis tool [26].

### Immunoblotting Analysis

Rabbit polyclonal-CagA, VacA, and UreA antibodies (Santa Cruz Biotechnology) were used to probe CagA, VacA, and UreA levels respectively, in *hpyAVIBM* deletion mutant and wild-type strains. Horseradish peroxidase-conjugated goat anti-rabbit IgG was used as secondary antibody (Bangalore Genei). Blots were developed with the ECL Plus Western blot reagents (Amersham Pharmacia) according to manufacture’s instructions. Densitometry was performed on scanned immunoblot images using the ImageJ gel analysis tool [26].

### IL-8 Assay

The strains were cultured on Brain Heart Infusion agar plates containing 7% sheep blood/horse serum for 3 days at 37°C under microaerobic conditions. Bacteria were harvested from 24 hrs grown culture and resuspended in phosphate-buffered saline (PBS). The bacteria concentration was estimated by nephelemetry and the suspension was centrifuged 15 mins at 2000 *g*. The supernatant was discarded and the pellet was resuspended in RPMI 1640 containing 10% FBS in order to obtain 5×10^8^ bacteria/mL. This suspension was used to infect the cell culture. AGS cells (ATCC CRL 1739, a human gastric adenocarcinoma cell line) were cultured in RPMI 1640 (HiMedia) medium supplemented with 10% FBS (Invitrogen, UK). They were grown for 3 days at 37°C, under 5% CO_2_. The cells were trypsinized (Gibco BRL), microscopically enumerated, and distributed in a 24-well microtiter plate at a final concentration of 1×10^5^ cells/mL (1 mL/well). The microtiter plate was incubated 24 hrs at 37°C prior to infection by 1 mL of the *H. pylori* suspension. A negative control (RPMI alone) was taken. All samples were tested in duplicate. Infected AGS cells were incubated for 8 hrs at 37°C. The medium was removed and centrifugated at 13000 *g* for 20 mins in order to remove the bacteria and the cell fragments. The supernatant was frozen prior to IL-8 measurement by ELISA. IL-8 measurement was performed using the specific ELISA kit provided by Amersham Biosciences (Interleukin-8(h) IL-8) ELISA Biotrak™ System) according to the manufacturer’s instructions.

### LPS Purification and Profiling

Equal number of mutant and wild-type *H. pylori* cells were harvested by centrifugation and washed once in PBS and once in PBS supplemented with 0.15 mM CaCl_2_/0.15 mM MgCl_2_. LPS was extracted from each sample as explained earlier [27]. The purified LPS was separated by SDS-PAGE and gel was stained with silver as explained earlier [28].

### Methylation Assay

All methylation assays were done to check the incorporation of tritiated methyl groups into DNA as described earlier [17].

### Natural Transformation

The *H. pylori* cells (wild type and mutant) to be transformed were grown on BHIA plates with 7% horse serum for 36 hrs and then harvested into 1 ml of PBS pH 7.4, centrifuged at 2000 g for 5 min, and the pellet resuspended in 200 µl of PBS. Each transformation mixture, consisting of 100 µl of recipient cells (∼10^6^ cells) and 10 µl (at 10 ng/µl) of plasmid DNA, was incubated on ice for 30 min. Then the mixture was spotted on BHIA plate and plates were incubated for 24 h at 37°C in a 5% CO_2_ atmosphere. The transformation mixture then was harvested into 1 ml of PBS, and appropriate serial dilutions inoculated to both BHIA (non-selective) and antibiotic (selective) plates with 15 mg/ml chloramphenicol and incubated for 4 days at 37°C in a 5% CO_2_ atmosphere. The number of colonies of transformants and total viable cells were counted and the transformation frequency was calculated as the number of chloramphenicol-resistant colonies per microgram of plasmid DNA per recipient CFU. Each experiment was repeated thrice with two independent biological replicates.

## Results and Discussion

Genome sequencing and analysis of a number of strains have shown a high degree of variation that exists in *H. pylori* [29]. To investigate the possible role(s) of *hpyAVIBM* in the physiology of *H. pylori,* we have selected two unrelated strains namely, SS1 and AM5. While SS1 is a mouse colonizing strain [30], AM5 is a clinical Indian strain isolated from a patient with duodenal ulcer [31].

### *hpyAVIBM* is a Phase Variable C^5^ Cytosine Methyltransferase

**Figure 1:**
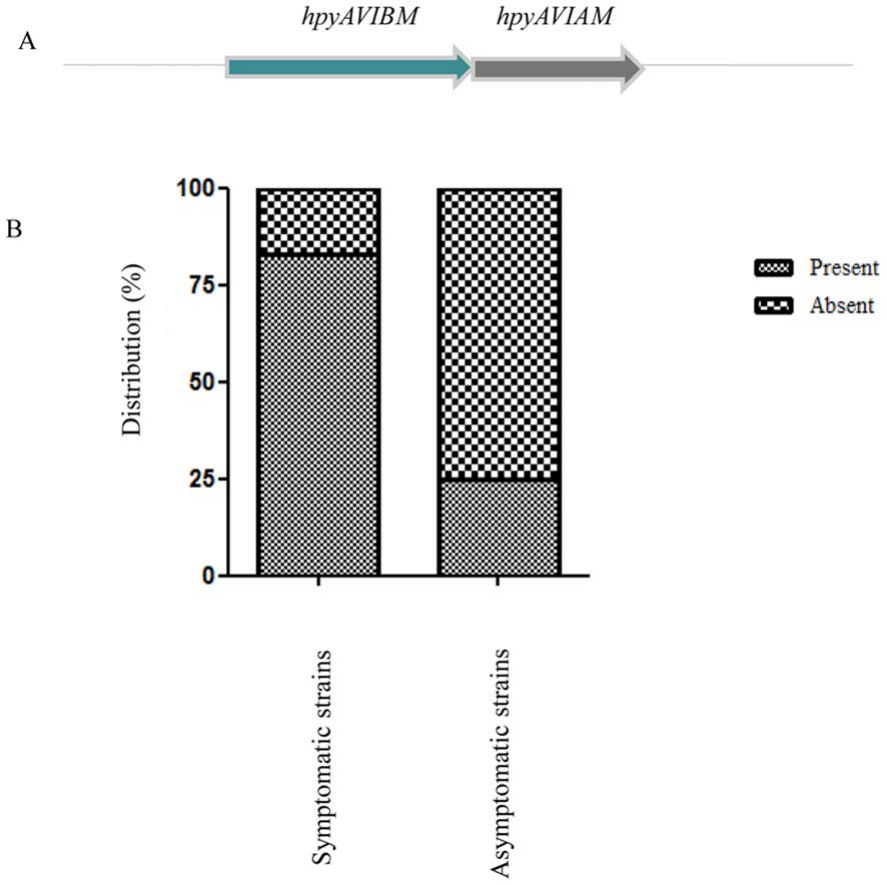
*hpyAVIAM* and *hpyAVIBM.* (**A**) Schematic presentation of operon coding *hpyAVIAM* and *hpyAVIBM*. (**B**) Distribution of *hpyAVIBM i*n symptomatic and asymptomatic strains. P<0.0001.

**Figure 2:**
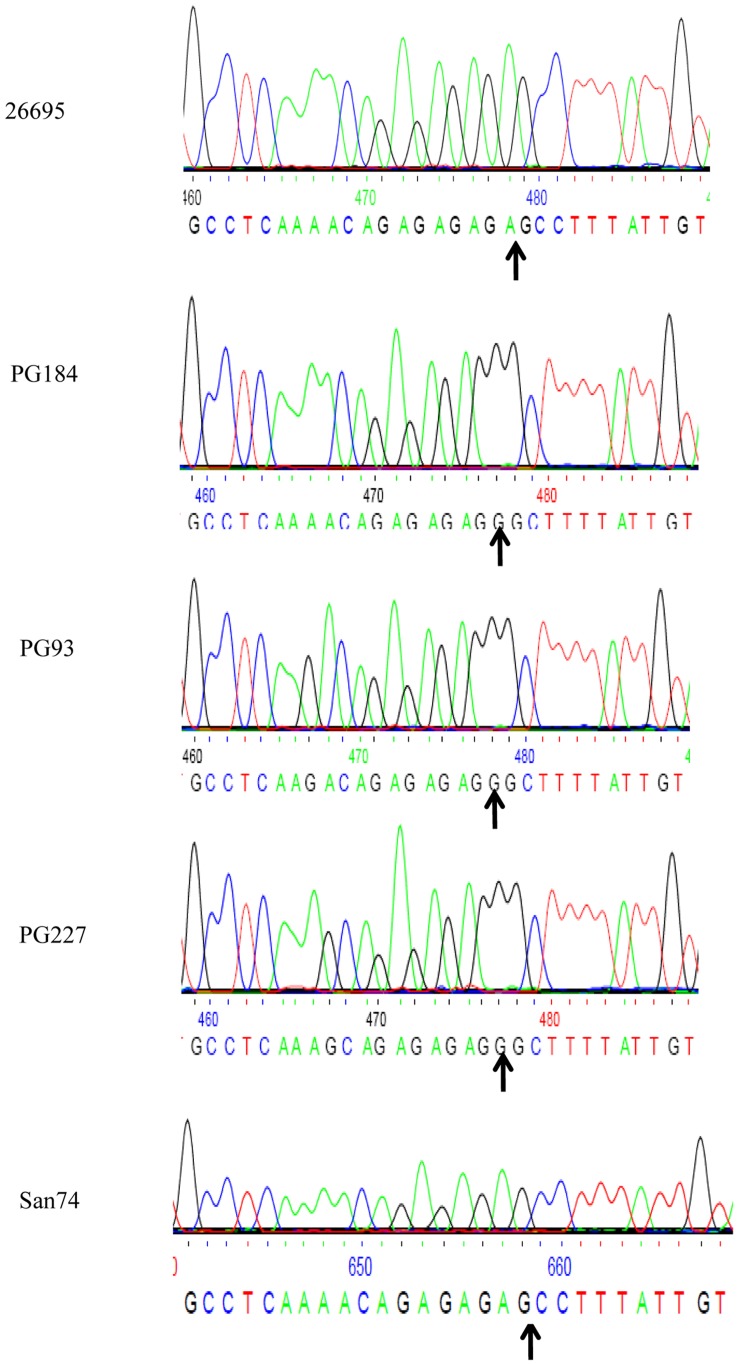
Variation in dinucleotide repeats in *H. pylori* clinical isolates. Arrow indicates the substitution/deletion mutation.

*hpyAVIAM* and *hpyAVIBM* are two solitary methyltransferases (MTases) in *H. pylori* strain 26695 (Fig. 1A) coded by a single mRNA [22], [32]. The presence of *hpyAVIBM* alleles was studied in different *H. pylori* strains isolated from 75 adult patients of both sexes with a diagnosis of duodenal ulcer (DU) on the basis of endoscopic examination of the stomach and duodenum, and 30 adult healthy volunteers of both sexes who had no gastritis or dyspeptic syndromes [31], [33]–[34]. PCR was done using two sets of primers, 1 & 2 and 3 & 4 (Table S1) designed from the most conserved regions of *hpyAVIBM*. It was observed that *hpyAVIBM* is present in 83% of the symptomatic strains and surprisingly, only in 25% of asymptomatic strains (Fig. 1B). *hpyAVIBM* allele was sequenced from a number of isolates to determine the number of AG repeats in the open reading frame. The presence of AG repeats in *hpyAVIBM* makes it a candidate for phase variation, which is a reversible switching between the phenotypes [20]. Any alteration in the number of repeats because of contraction or expansion can lead to a frame shift mutation in *hpyAVIBM*, thus resulting in inactivation of the MTase. In strain San 74, because of deletion, *hpyAVIBM* has 4 AG repeats compared to 5 AG repeats in strains 26695 and HPAG1 thus, causing the translation of a truncated protein (Fig. 2). Interestingly, strains, PG184, PG93, and PG227 where *hpyAVIBM* is in frame, have only four AG repeats compared to five present in strain 26695 (Fig. 2). Sequence analysis showed that the decrease in AG repeats is because of the substitution mutations and not due to phase variation. With the increase in the number of repeats, frequency of phase variation increases and vice versa [20]–[21]. Thus, decrease in the number of AG repeats in strains PG184, PG93, and PG227 possibly makes *hpyAVIBM* less prone to phase variation [20].

### Deletion of *hpyAVIBM* has Differential Effects on Unrelated Strains

*hpyAVIBM* was PCR amplified from genomic DNA of *H. pylori* strains AM5, 26695, and SS1, cloned in expression vector and the proteins purified to near homogeneity (Fig. S1A) [32]. HpyAVIBM is known to recognize CCTC and methylates the first cytosine [13]. HpyAVIBM MTase from these strains was active and inhibited in the presence of sinefungin (Fig. S1B). HpyAVIBM methylated the first cytosine in 5′ CCTC 3′ recognition sequence (data not shown). In order to understand the role of cytosine methylation by HpyAVIBM MTase, a knockout strain of *hpyAVIBM* was constructed in two distinct *H. pylori* strains - AM5and SS1. Deletion of *hpyAVIBM* was confirmed by PCR (Fig. S2). The overall expression profile was compared between the wild-type and knockout strains of AM5and SS1. Comparative expression profile analysis showed that a number of genes with altered expression encoded for the components involved in motility, pathogenesis, outer membrane proteins (OMPs), restriction-modification systems, and lipopolysaccharide (LPS) synthesis (Table 1). Alteration in the transcript levels of genes involved in different metabolic pathways were also observed (Tables 1 and S2). Interestingly, the deletion of *hpyAVIBM* in different *H. pylori* strains had different effects on gene expression. The differential effect of *hpyAVIBM* knockout in different strains could be because of different genetic background of the strains. When the distribution of CCTC sites in *H. pylori* strains 26695, J99 and HPAG1 was analyzed, it was observed that positioning of CCTC sites differed from strain to strain (http://rsat.ulb.ac.be/rsat/) and this could be the reason for the difference in the effect of deletion of *hpyAVIBM*. Deletion had differential effect on the expression of around 400 transcripts between two strains (Table S2). In addition, RAPD analysis of strains AM5, SS1, 26695, J99, PG227 and PG225 was done by using the primers having GAGG sequence at the 3′ end. These primers (primers 35–38, Table S1) amplified the DNA sequences between two CCTC sequences. A differential RAPD profile was observed for all the strains, suggesting the differences in the distribution of CCTC in three genomes (Fig. S3). It has been shown earlier that deletion of a house keeping gene *ppk1* in unrelated strains can have differential effects in motility, growth and susceptibility to metronidazole [23]. It has been shown earlier in an *in vitro* experiment, that methylation by HpyAVIBM can alter the DNA- protein interactions [35]. It could be possible that methylation by HpyAVIBM alters the expression of genes in *H. pylori* by a similar mechanism.

**Table 1:** Comparative transcriptomics of *H. pylori* wild type vs *hpyAVIBM* deletion mutant of strains AM5 and SS1.

	AM5		SS1	
Gene No.	Microarray analysis Fold change	RT PCR	Microarray analysis Fold change	RT PCR
**Outer membrane protein**				
HP0009(Omp 1, HopZ)	−2.98	ND	−5.06	ND
HP0079(Omp 3, HorA)	2.6	ND	1.94	ND
HP0127(Omp 4, HorB)	3.7	ND	–	ND
HP0229(Omp 6, HopA)	−3.3	ND	–	ND
HP0472(Omp 11, HorE)	−3.03	−2.9	2.5	1.8
HP0896 (Omp19,BabB)	2.67	2.9	3.06	2.2
HP1243 (Omp28,BabA)	2.80	3.1	4.3	2.5
HP1395(Omp 30, HorL)	2.9	ND	1.54	ND
HP0638 (oipA)	1.7	ND	–	–
**Motility**				
HP0713 (FliR)	5.4	3.5	1.7	2.8
HP0714 (RpoN)	−1.2	−1.4	2.7	1.4
HP0752 (FliD)	−0.3	ND	–	–
HP0753 (FliS)	−0.07	−1.1	−1.09	ND
HP0815 (MotA)	−0.33	–	−1.1	ND
HP0906 (FliK)	−1.9	−1.6	1.4	1.5
HP1119 (FlgK)	−1.04	ND	–	ND
**Pathogenicity**				
HP0547 (CagA)	2.26	2.9	4.7	2.4
HP0887 (VacA)	2.7	4.5	6.9	2.3
HP0315(VapD)	4.3	4.3	3.1	1.5
HP1399(Arginase)	3.05	5.5	5.1	4.5
**LPS biosynthesis**				
HP0379(FutA)	3.47	3.2	−2.8	−2.8
HP0651(FutB)	5.35	5.7	−0.156	−0.12
HP0093–94(FutC)	−3.05	−3.0	1.8	1.7
HP1105	4.4	ND	12.1	ND
HP0511	6.4	ND	10.0	ND
HP0217	5.61	ND	5.7	ND
HP0326	2.39	ND	2.3	ND
HP0327	2.5	ND	1.2	ND
HP0102	4.62	ND	2.6	ND
**Restriction Modification system**				
HP0091	4.4	ND	5.05	ND
HP0092	2.88	ND	1.1	ND
HP0262	6.26	ND	5.5	ND
HP0263	3.25	ND	4.2	ND
HP1366	3.36	ND	10.05	ND
HP1367	3.02	ND	9.4	ND
HP1368	3.38	ND	5.7	ND
HP0848	5.01	ND	5.9	ND
HP0849	3.3	ND	5.8	ND
HP0850	4.25	ND	9.8	ND
HP1208	6.7	ND	5.6	ND
HP1209	11.4	ND	13.8	ND
HP0909	3.9	ND	–	ND
HP1522	–	ND	–	ND
HP0369	2.46	ND	–	ND

The genes listed are either down- or up-regulated in the *hpyAVIBM deletion* mutant of *H. pylori* strains AM5 and SS1. The identity of each gene is indicated by the Locus name as annotated in the *H. pylori* strain 26695 genome. The average ratio presented is the mean of mutant/wt ratio. P value <0.005.

ND: not determined.

- : not significant.

**Figure 3:**
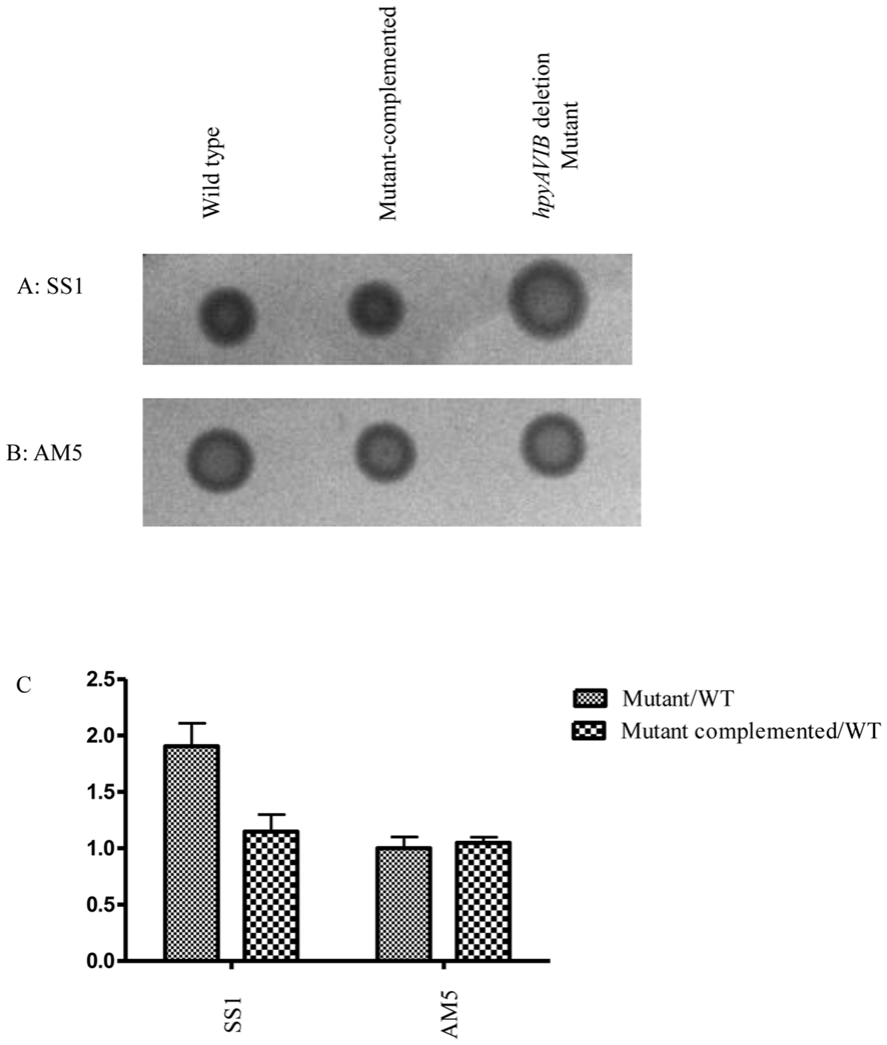
Motility assay. The photograph shows a BHI 7% FBS soft agar plate after 4 days of incubation. (**A**) SS1 (**B**) AM5 (**C**) Graph showing relative change in the diameter of mutant vs wild-type strain.

### Transcriptomic Analysis of HpyAVIBM Methylation Dependent Expression of Outer Membrane Protein Genes

In *H. pylori*, the outer membrane mediates the interaction of the bacterium with its surroundings. Comparative analysis of complete genome sequences has confirmed the presence of a large number of integral outer membrane proteins (OMPs) that represent around 4% of each strain’s coding potential [5], [36]. During infection, proteins present on the outer membrane of *H. pylori* are assumed to be altered in such a way that recognition by the host immune system is minimal [37]. When *hpyAVIBM* was deleted in different strains of *H. pylori*, changes in the expression profile of a number of OMPs were observed. Microarray analysis coupled with RT-PCR showed increase in the transcript levels of *babA* and *babB* in AM5*ÄhpyAVIBM* and SS1 *ΔhpyAVIBM* (Fig. S4 and S5). *babA* and *babB* have a vital relation with adherence, as *babA* binds to Lewis b antigen, which is expressed in the human gastric mucosa of most individuals [38]. Interestingly, *omp11* transcript levels were increased in SS1 mutant strains, and decreased in AM5 mutant strain (Table 1, Figs. S4 and S5). It has been shown that *omp11* is antigenic [39]. Bacterial adherence is an important contributor to the extent of infection and virulence [40] and the expression of outer membrane proteins varies from strain to strain [41]–[42]. The mechanism for variable expression of OMPs is not very well understood. Our results suggest that strain-specific methylation pattern could be one of the reasons for variation in the OMPs expression. This variability can result in a population comprising different sub-populations having different outer membrane protein patterns, thus having differential interaction with the host. By controlling the host-bacterial interaction, OMPs also regulate the severity of infection. It has been postulated that the metastability and heterogeneity in adhesin proteins can play a significant role in the bacterial fitness within a host [43].

**Figure 4:**
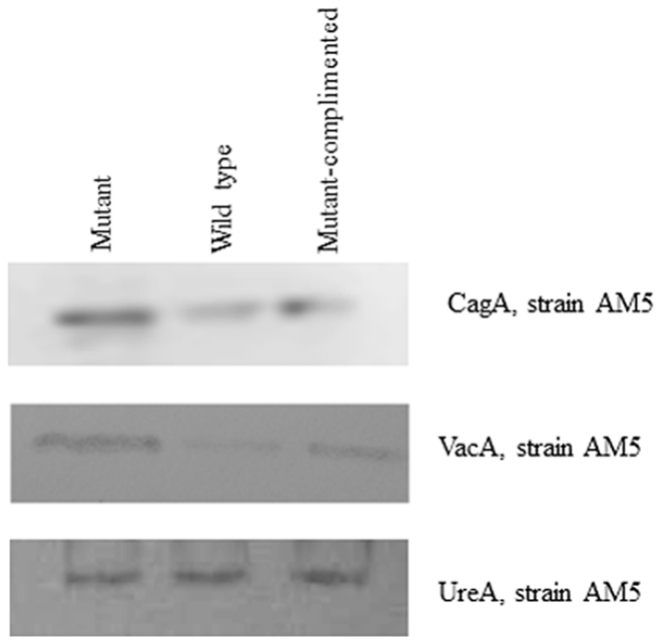
Western blotting for CagA and VacA protein levels in *H. pylori* strainsAM5 and AM5Δ*hpyAVIBM*.

### *H. Pylori* Strain SS1*ΔhpyAVIBM* is More Motile than the Wild-type

Motility is one of the important factors for colonization by *H. pylori*. The flagellum helps bacterium to move in the highly viscous mucous layer of the gastric epithelium. Flagellar synthesis involves sequential assembly of more than 40 flagellar proteins. Comparisons between wild-type strains and their respective *hpyAVIBM* deletion mutants showed the change in the expression of a number of flagellar genes like, *rpoN, fliR, fliD, fliS, motA, fliK* and *flgK*. Intriguingly, increase in the expression of *rpoN* (sigma 54) transcript was observed in *hpyAVIBM* deletion strain of SS1 while there was a decrease in AM5*ÄhpyAVIBM* strain. RpoN controls the expression of middle flagellar genes (class II), including the expression of FliK which is the hook length protein [44]. Change in the expression of *fliK* was similar to that observed for *rpoN*. Moreover, expression of *flgK* (Flagellar hook-associated protein 1) varied similarly, that is increased in SS1 *ÄhpyAVIBM* and decreased in AM5 *ΔhpyAVIBM* (Table 1, Figs. S4 and S5). The phenotypes of three mutants were analyzed by motility assay as described in materials and methods (Fig. 3A–C). It was observed that *hpyAVIBM* deletion resulted in an increase in motility in mutant strain of SS1 but no change was observed in the motility of AM5 mutant (Fig. 3B–3C). Wild-type phenotype was restored when the mutants were complemented with *hpyAVIBM* cloned in pHel3 with its own promoter. It has been shown earlier that post translational modification like glycosylation of flagellin is critical for motility [45]. It is possible that alteration in motility could be because of change in glycosylation pattern of flagellin proteins. Flagellar motility is a critical component for successful gastric colonization and suborgan localization within the stomach by the ulcer-causing bacterium *H. pylori*. Modulation of motility is important for *H. pylori* as it drives the bacteria towards beneficial conditions and away from harmful ones.

**Figure 5:**
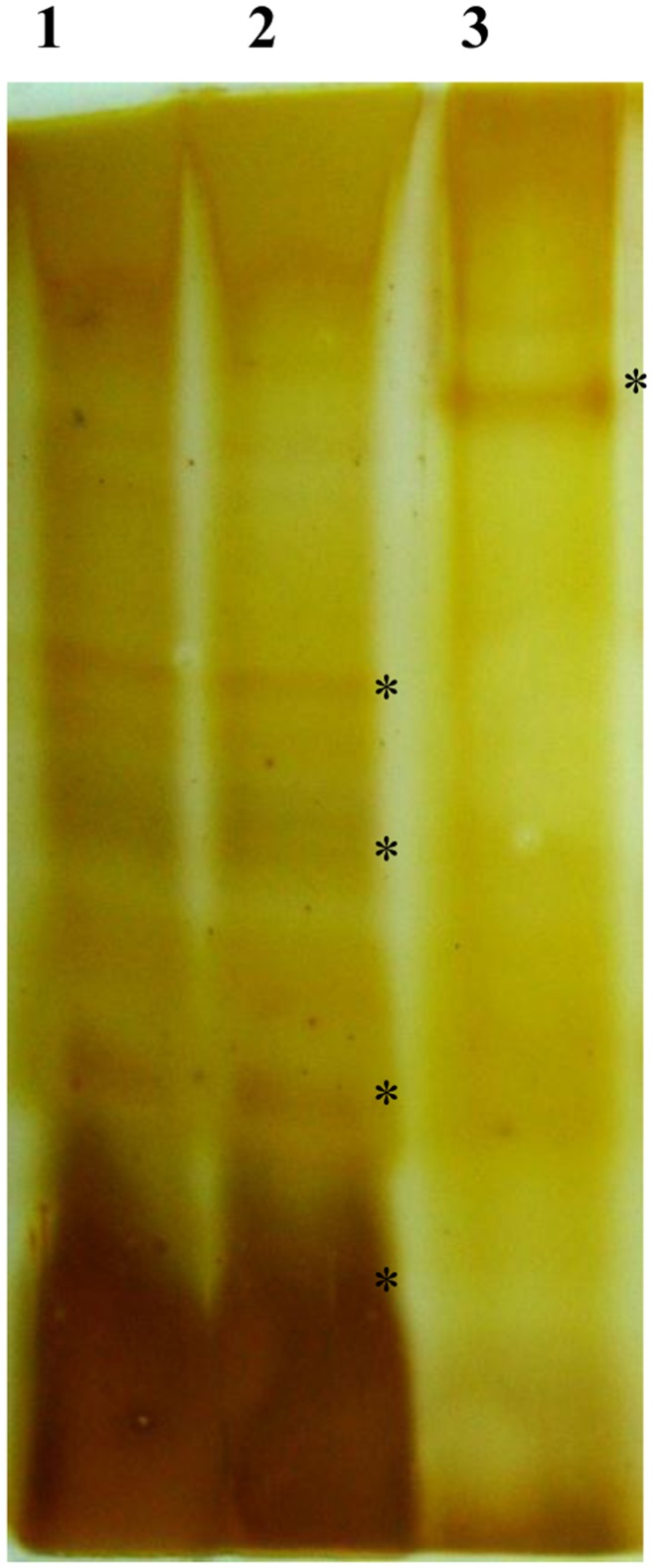
LPS profiles of *hpyAVIBM* deletion mutant and wild-type strains. Lane 1: wild-type strain AM5, lane 2: *hpyAVIBM* deletion mutant AM5 complemented with *hpyAVIBM* from strain 26695, lane 3: *hpyAVIBM* deletion mutant. * highlights the bands different between wild type and mutant.

### *hpyAVIBM* Suppresses the Expression of *cagA*, *vacA*, *vapD* and *hp1399* (Arginase) in *H. Pylori* Strain AM5

CagA and VacA are the most immunogenic *H. pylori* proteins that are responsible for different pathogenic properties of *H. pylori* strains [40]. They are responsible for causing morphological changes, vacuolization and membrane channel formation in epithelial cells [46]–[47]. A 2.9 -fold increase in the transcript of *cagA* was observed in AM5 mutant strain which was confirmed by RT-PCR (Fig. S4) and Western blotting using CagA specific antibodies (Fig. 4). AM5 and SS1 mutants showed a significant increase in the transcript of *vacA* (Table 1). *vapD* is a strain-variable gene and is present in about 60% of *H. pylori* strains. *vapD* gene is closely related to the gene encoding virulence-associated protein D of *Dichelobacter nodosus* [48]. Around 4 fold increase was observed in AM5 mutant compared to 3 fold increase in SS1 mutant strain for the transcript of *vapD*. For a successful pathogen like *H. pylori* it is very important to regulate the function of its virulence factors in order to avoid or suppress the host immune system. CagA and VacA on one hand are responsible for inducing proinflammatory response by the host and on the other hand they suppress T cell function [40]. Arginase is another protein, which helps the bacteria to overcome host defenses [49]. More than 4- fold increase was observed in the transcript levels of gene coding for arginase in *hpyAVIBM* deletion strains of SS1 and AM5 (Fig. S4 and S5). These data suggest that the regulation of virulence factors by *hpyAVIBM* may play an important role in helping bacterial cells to cope with a changing environment and thus, adds an extra dimension to the host-pathogen interaction. The role of adenine methylation in regulation of virulence factors is well established in a number of pathogens like *Salmonella* sp. and *Neisseria* sp. [7], [10]. Our results clearly indicate a possible role of cytosine methylation in the regulation of virulence.

**Figure 6:**
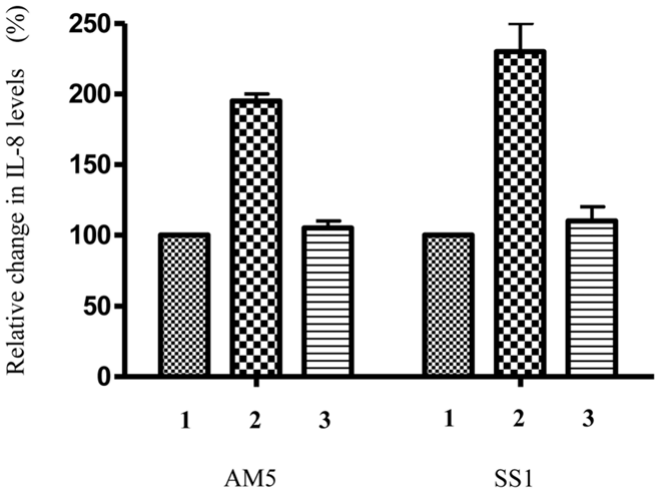
*hpyAVIBM* deletion enhances IL-8 production in AGS cell lines. (1) *H. pylori* strain AM5/SS1 (2) *H. pylori* strain AM5*ΔhpyAVIBM/*SS1*ΔhpyAVIBM* (3) *hpyAVIBM* deletion mutant AM5/SS1 complemented with *hpyAVIBM* from strain 26695.

### *H. Pylori* Strain AM5*ΔhpyAVIBM* has an Altered LPS Profile Compared to the Wild-type

The most variable features of *H. pylori* are the structures present on its surface. Changing these surface molecules is a way to evade the immune system or to alter the expression of characteristic molecules important for interaction with host cells [50]. A major surface structure of Gram-negative bacteria is lipopolysaccharides (LPS). Interestingly, in *H. pylori* LPS is modified by addition of fucose sugar. Fucosylation mimics Lewis antigens, structures found on human erythrocytes and epithelial cells. Three fucosyltransferases namely, FutA, FutB, and FutC are responsible for the addition of fucose sugars [27]–[28], [51].

**Figure 7:**
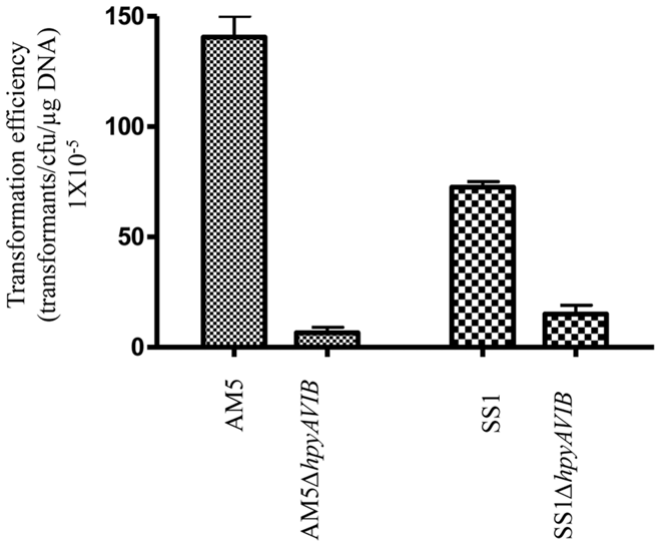
Natural transformation efficiency of *H. pylori* strains AM5 and SS1 and their respective *hpyAVIBM* deletion mutant strains. Values are calculated as transformants/cfu/mg DNA. P<0.0001.

It has been shown that expression of the three fucosyltransferases in *H. pylori* is regulated via slipped-strand mispairing in intragenic polyC tract regions, resulting in different reading frames. ON/OFF switching of these genes in different combinations gives rise to a mixed population with different Lewis glycosylation patterns [27]–[28], [51]. An increase in the transcript levels of *futA* and *futB*, while a 3- fold suppression in the expression of *futC* was observed in AM5 mutant strain (Table 1, Fig. S4 and S5). In SS1 mutant, an increase in *futC* transcript levels was observed. FutA and FutB are responsible for the synthesis of Lewis x antigen and FutC activity yields Lewis y antigen [27]. A shift in the expression of different fucosyltransferases can alter the membrane topography and in turn interaction with the host [28]. Other than change in the expression of fucosyltransferases, alterations in the expression profile of many other genes involved in LPS biosynthesis were observed (Table 1). Total LPS was isolated from mutant and wild-type *H. pylori* strains AM5 and 26695 as explained earlier [27] and the LPS profiles were compared. A significant difference was observed in the LPS profiles of AM5*ΔhpyAVIBM* and wild-type strains whereas the LPS profile of mutant complemented with *hpyAVIBM* was similar to the wild-type (Fig. 5).

**Figure 8:**
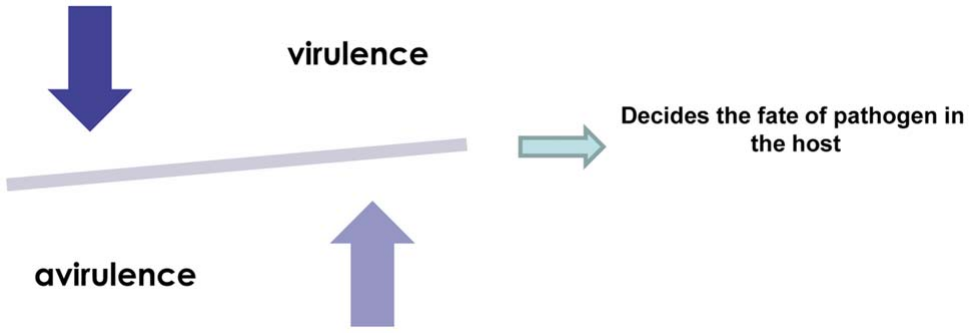
Survival of a pathogen in host depends upon its ability to modulate the balance between virulence and avirulence.

### *hpyAVIBM* Knockout Enhances the IL-8 Production in AGS Cell Line

An inflammatory response is the key pathophysiological event in *H. pylori* infection. It has been suggested that cytokines mediate the mucosal inflammation caused by *H. pylori* [52]. IL-8 is a potent chemoattractant and an activator of neutrophils and is thought to play a central role in gastric mucosal injury caused by *H. pylori* [53].

It has been shown that CagA, VacA, OMPs (OipA) and LPS have an effect on IL-8 production from host cells [40], [52]–[54]. In order to see the effect of *hpyAVIBM* deletion on the ability of *H. pylori* strains to induce IL-8 production in AGS cell line, mutant and wild-type cells were co-cultured with AGS cell line separately and IL-8 measurement was performed as explained in materials and methods. As can be seen from figure 6, AM5*ΔhpyAVIBM* and SS1*ΔhpyAVIBM* were able to induce IL-8 production 2- fold higher than the wild-type (Fig. 6). High IL-8 production is in accordance with the increase in the expression in *cagA, vacA* and OMPs like *babA babB* and *oipA* in mutant strains (Table 1, Figs. S4 and S5).

### *hpyAVIBM* Deletion Hinders the Transformation Efficiency of the Mutant Strains

*H. pylori* displays natural competence for genetic transformation [55]. High natural competency is the basis for horizontal gene transfer and subsequent generation of a high degree of genetic diversity that exists in *H. pylori*. On the other hand genomic sequences of *H. pylori* strains have revealed that the bacterium contains an abundance of restriction and modification genes. It has been demonstrated R-M systems in *H. pylori* are a barrier to interstrain plasmid transfer [56]. By modulating the activity of different R-M systems, *H. pylori* in turn can regulate its stringency to take up DNA from the environment. *H. pylori* has a number of R-M systems that are prone to phase variation. Phase variation can switch ON or OFF (21) the R-M system and thus can regulate the extent of horizontal gene transfer. Microarray analysis revealed that several R-M systems get strongly up-regulated in *hpyAVIBM* deletion strains especially in AM5*ΔhpyAVIBM* strain, which includes a number of Type II R-M systems (Table1). Elevation in the activity of R-M systems can pose a strong barrier to the transformation of DNA. pHel3 plasmid was used to check the transformation efficiency of mutant and wild-type strains. It was found that AM5*ΔhpyAVIBM* shows 50-fold decrease in the transformation efficiency as compared to the wild-type, possibly because of increased activity of a number of restriction enzymes. Similar effects were observed for SS1*ΔhpyAVIBM* mutants as it showed 10–20 fold decrease in the transformation efficiency (Fig. 7). The ability of *H. pylori* to take up DNA from the environment plays a critical role in the creation of variability. It is known that mismatch repair proteins are another set of factors that can influence competency. However, in the absence of most of the MMR proteins in *H. pylori*, R-M systems could be playing a significant role in transformation [57]. Modulation in the expression of the genes coding for R-M systems can influence the natural competency of *H. pylori*, thus affecting the rate of generation of genetic variability.

The present study shows the multidimensional effects of cytosine methylation on gene expression in *H.pylori*. The addition of a methyl group is a significant modification of a cytosine base which in turn can affect the interaction between DNA transacting proteins and DNA. It was shown earlier in an *in vitro* experiment, that methylation of the promoter of *AOXI* encoding alcohol oxidase by HpyAVIBM hinders the binding of Mxr1p (methanol expression regulator 1) which, functions as a key regulator of methanol metabolism in the methylotrophic yeast *Pichia pastoris* [35].

It should be noted here that AOXI promoter contains HpyAVIBM recognition sequence. Comparative genome analysis has shown that a high degree of variation exists in *H. pylori*. Nucleotide sequences of different *H. pylori* strains exhibit an extremely high level of variation but the majority of nucleotide changes are synonymous substitutions [58]. This would result in comparatively less variation at the protein level. However, differences at the nucleotide level would result in differential distribution of recognition sequence of a R-M system between *H. pylori* strains. Differential distribution of recognition sites (of a MTase) between strains would result in different methylation pattern which in turn result in variable gene expression profile thus, adding another dimension to the variability function in *H. pylori*. *H. pylori* has a highly plastic genome [59]. *H. pylori* continuously alters its genome by point mutations and interstrain recombination to cope up with the ever-changing micro-environment. Our data indicates that DNA methylation by HypAVIBM MTase could be playing a critical role in modulating the expression of genes involved in virulence and its interaction with the host. It could be possible that methylation by HpyAVIBM alters the interaction between the regulatory factors and cognate recognition sites on the promoters of target genes. It was observed that a number of genes involved in virulence and colonization like CagA, VacA, several outer membrane proteins and genes controlling motility were up-regulated in the *hpyAVIBM* knockout strains. Thus, the wild-type and the knockout strains would interact differentially with the host. The knockout strain induced strong immune response in the AGS cell line as monitored by high IL-8 induction, because of the up-regulation of a number of virulent factors. For a successful pathogen like *H. pylori*, it is very important to maintain a balance between virulence and avirulence (Fig. 8). A virulent pathogen can provoke a strong host immune response and this can remove the bacteria from the system. *H. pylori* must have developed mechanisms by which it can modulate its virulence according to the need for a successful survival in the host like mimicking Lewis antigens [27]–[28],[51]. It is quite extraordinary that in spite of many immunogenic virulent factors like CagA and VacA *H. pylori* is able to survive in most of the hosts without triggering a strong host immune system. Additionally, it has developed mechanisms to neutralize host immune responses, and proteins like catalase and arginase could be playing a significant role in this tussle between host and pathogen. An extra dimension in this interaction is added by the fact that *H. pylori* strains are genetically diverse and a single host can have more than one strain. In addition, it was observed that the effects of methylation differ from strain to strain, thus creating more variability in the habitat. For an organism like *H. pylori* with a limited host range and a small genome coding for very few number of regulatory proteins, controlling gene expression by differential methylation is a likely mechanism to cope up with change in the host environment.

## Supporting Information

Figure S1 **Purification and methylation activity.** (A) Purification of HpyAVIBM. Lane 1: marker, purified HpyAVIBM from strain lane 2∶26695, lane 3: SS1, lane 4: AM5 (B) Methylation activity of HpyAVIBM homologs from strains 26695, SS1 and AM5 in the presence and absence of sinefungin (Sf).
(TIF)

Figure S2 **Screening of** ***hpyAVIBM*** **deletion mutant in** ***H. pylori*** **strains AM5 and SS1.** (**A**) Positioning of primers for the screening of *hpyAVIBM* deletion. (**B**) Screening of *hpyAVIBM* deletion mutant., Lane 1: wild-type *H. pylori* strain AM5, lane 2: *H. pylori* strain AM5*Δ hpyAVIBM,* lane 3: wild-type *H. pylori* strain AM5, lane 4: *H. pylori* strain AM5*Δ hpyAVIBM*, M: marker, lane 5: *H. pylori* strain SS1, lane 6: : *H. pylori* strain SS1*Δ hpyAVIBM*, lane 7: *H. pylori* strain SS1, lane 8: : *H. pylori* strain SS1*Δ hpyAVIBM*.
(TIF)

Figure S3 **RAPD analysis of H.** ***pylori*** **strains. Lanes 1-2∶26695, 3: SS1, 4: AM5, 5: J99, 6: PG227, 7: PG225.**
(TIF)

Figure S4 **Confirmation of transcriptional changes in selected genes in AM5Δ** ***hpyAVIBM*** **deletion mutant compared to wild type using RT PCR.** 16SrRNA was used as control. 1:Omp11, 2:Omp19, 3:Omp28, 4:HP0713(*fliR*), 5:HP0714(*rpoN*), 6:HP0753 (*fliS*), 7:HP0906 (*fliK*), 8:HP0547 (*cagA*), 9:HP0887 (*vacA*), 10:HP0315 (*vapD*), 11:HP1399, 12:HP0379 (*futA*), 13:HP0651 (*futB*), 14:HP0093 (*futC*).
(TIF)

Figure S5 **Confirmation of transcriptional changes in selected genes in SS1Δ** ***hpyAVIBM*** **deletion mutant compared to wild type using RT PCR.** 16SrRNA was used as control. 1:Omp11, 2:Omp19, 3:Omp28, 4:HP0713(*fliR*), 5:HP0714 (*rpoN*), 6:HP0753 (*fliS*), 7:HP0906 (*fliK*), 8:HP0547 (*cagA*), 9:HP0887 (*vacA*), 10:HP0315 (*vapD*), 11:HP1399, 12:HP0379 (*futA*), 13:HP0651 (*futB*), 14:HP0093 (*futC*).
(TIF)

Table S1 **Primers used in the study.**
(DOC)

Table S2 **Microarray analysis of** ***H. pylori*** **wild type vs hpyAVIBM deletion mutant of strains AM5 and SS1.**
(XLS)
