# Supplementary figures and images for: Comparative Transcriptomics of H. pylori Strains AM5, SS1 and Their hpyAVIBM Deletion Mutants: Possible Roles of Cytosine Methylation
Source: PLoS One. 2012 Aug 3;7(8):e42303. doi: 10.1371/journal.pone.0042303 (PMC3411764; doi:10.1371/journal.pone.0042303)

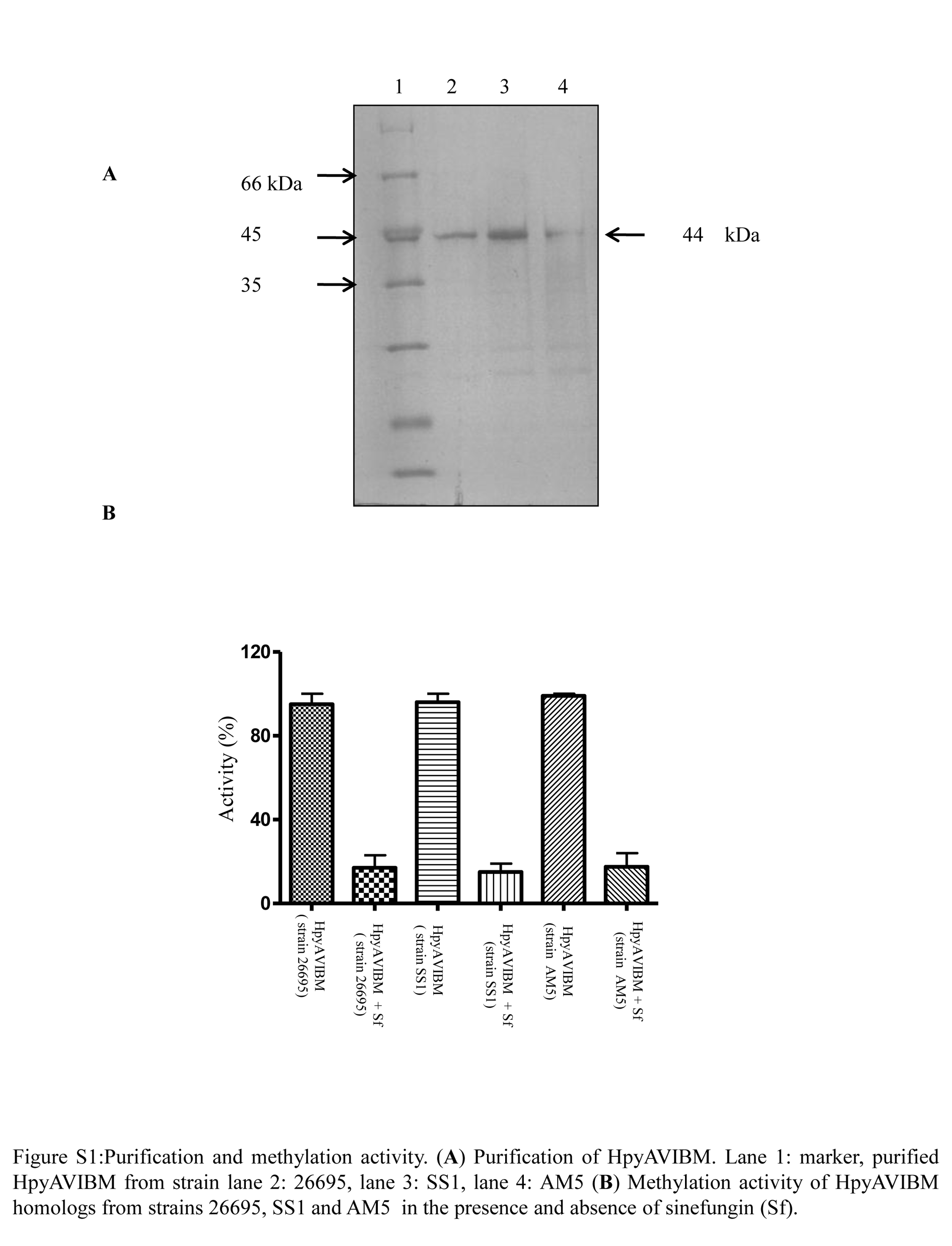

Supplement: Figure S1 — Purification and methylation activity. (A) Purification of HpyAVIBM. Lane 1: marker, purified HpyAVIBM from strain lane 2∶26695, lane 3: SS1, lane 4: AM5 (B) Methylation activity of HpyAVIBM homologs from strains 26695, SS1 and AM5 in the presence and absence of sinefungin (Sf). (TIF) [file pone.0042303.s001.tif]

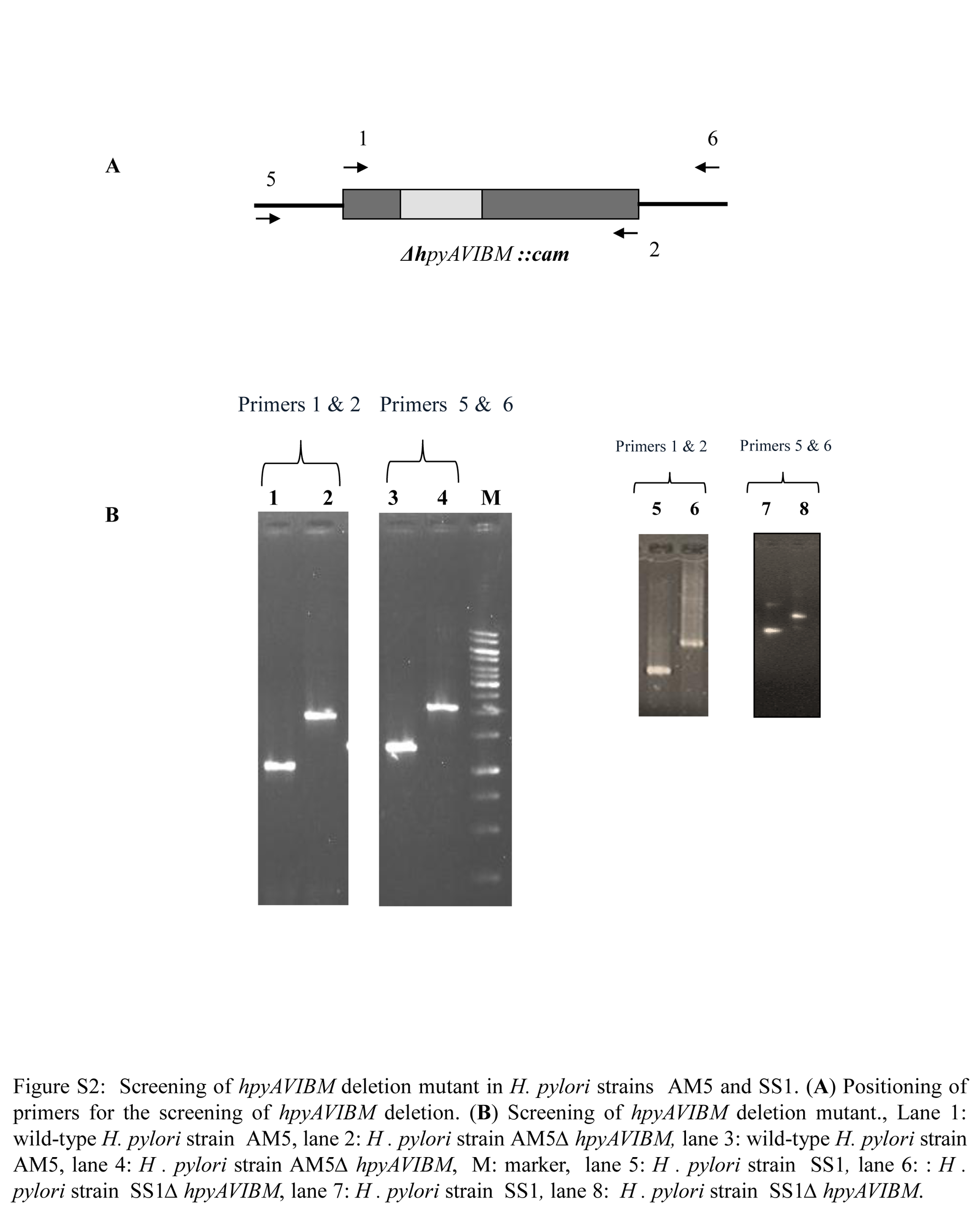

Supplement: Figure S2 — Screening of hpyAVIBM deletion mutant in H. pylori strains AM5 and SS1. (A) Positioning of primers for the screening of hpyAVIBM deletion. (B) Screening of hpyAVIBM deletion mutant., Lane 1: wild-type H. pylori strain AM5, lane 2: H. pylori strain AM5Δ hpyAVIBM, lane 3: wild-type H. pylori strain AM5, lane 4: H. pylori strain AM5Δ hpyAVIBM, M: marker, lane 5: H. pylori strain SS1, lane 6: : H. pylori strain SS1Δ hpyAVIBM, lane 7: H. pylori strain SS1, lane 8: : H. pylori strain SS1Δ hpyAVIBM. (TIF) [file pone.0042303.s002.tif]

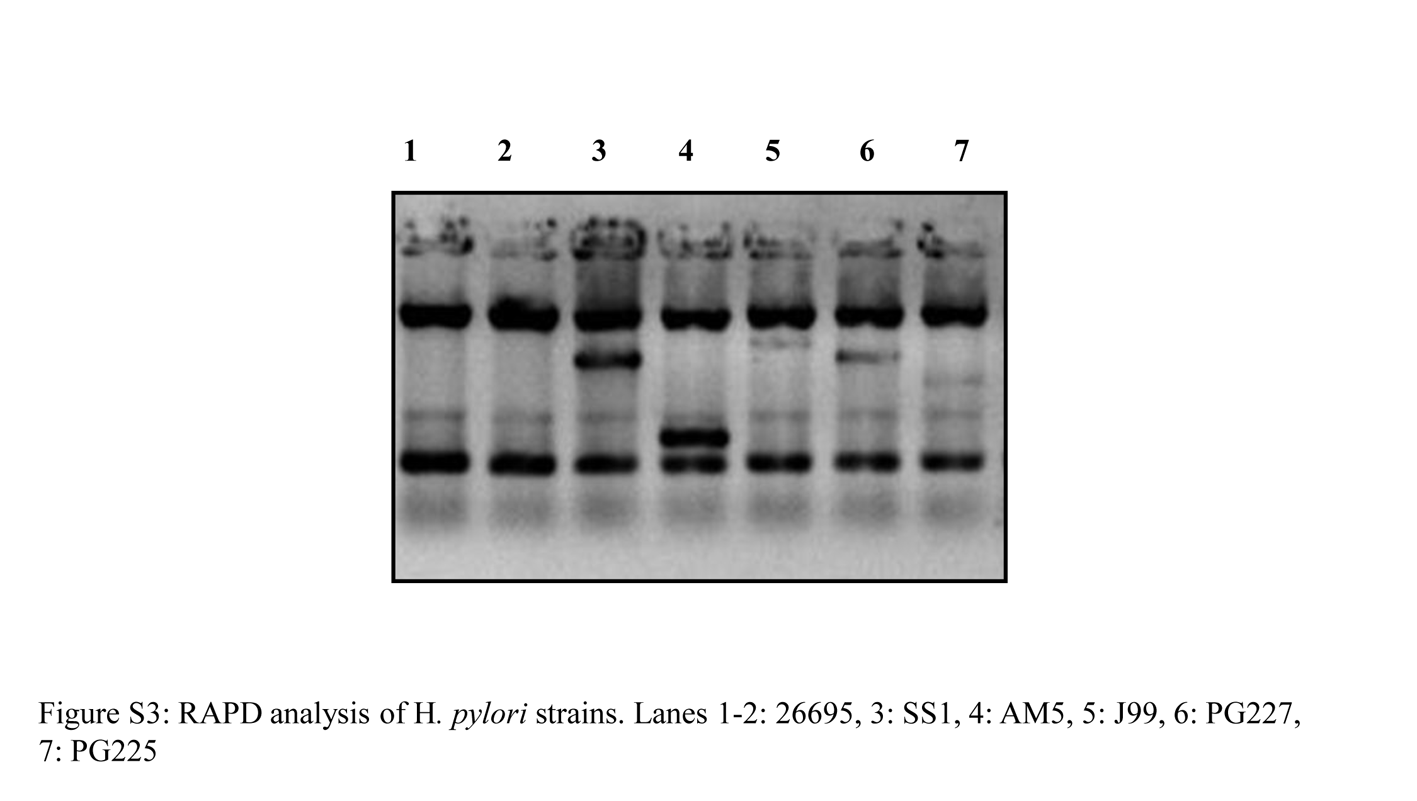

Supplement: Figure S3 — RAPD analysis of H. pylori strains. Lanes 1-2∶26695, 3: SS1, 4: AM5, 5: J99, 6: PG227, 7: PG225. (TIF) [file pone.0042303.s003.tif]

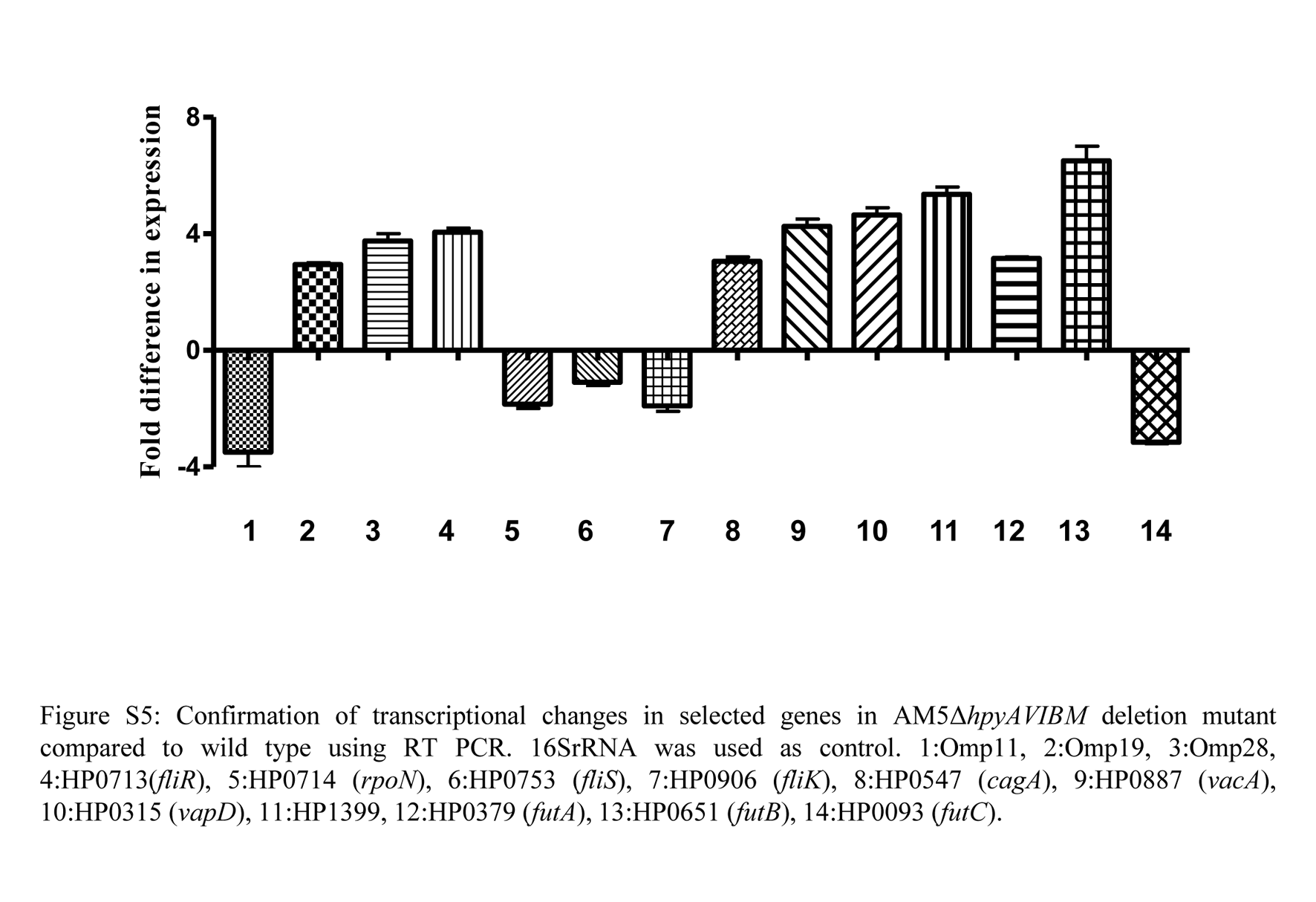

Supplement: Figure S4 — Confirmation of transcriptional changes in selected genes in AM5Δ hpyAVIBM deletion mutant compared to wild type using RT PCR. 16SrRNA was used as control. 1:Omp11, 2:Omp19, 3:Omp28, 4:HP0713(fliR), 5:HP0714(rpoN), 6:HP0753 (fliS), 7:HP0906 (fliK), 8:HP0547 (cagA), 9:HP0887 (vacA), 10:HP0315 (vapD), 11:HP1399, 12:HP0379 (futA), 13:HP0651 (futB), 14:HP0093 (futC). (TIF) [file pone.0042303.s004.tif]

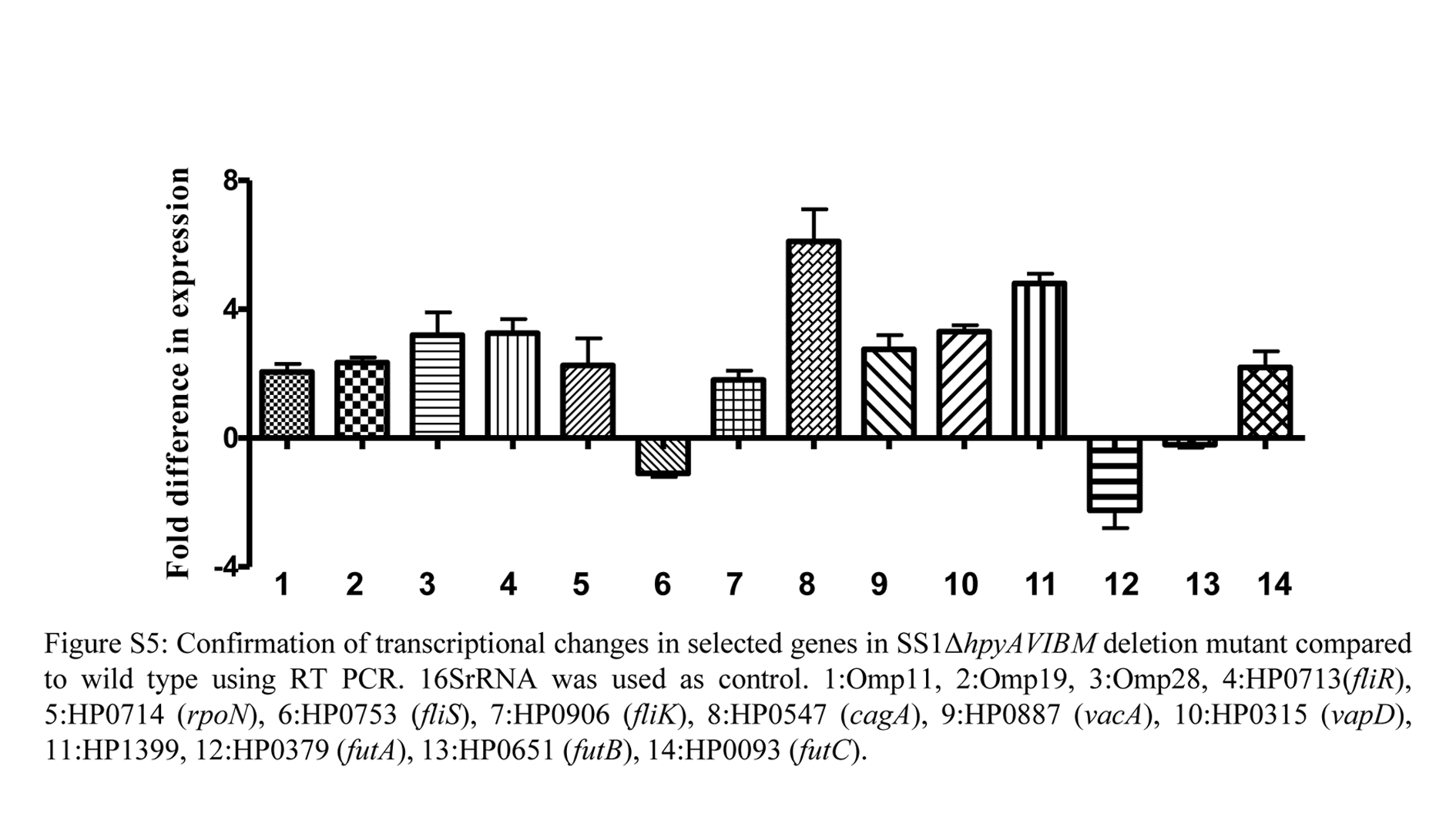

Supplement: Figure S5 — Confirmation of transcriptional changes in selected genes in SS1Δ hpyAVIBM deletion mutant compared to wild type using RT PCR. 16SrRNA was used as control. 1:Omp11, 2:Omp19, 3:Omp28, 4:HP0713(fliR), 5:HP0714 (rpoN), 6:HP0753 (fliS), 7:HP0906 (fliK), 8:HP0547 (cagA), 9:HP0887 (vacA), 10:HP0315 (vapD), 11:HP1399, 12:HP0379 (futA), 13:HP0651 (futB), 14:HP0093 (futC). (TIF) [file pone.0042303.s005.tif]
