# Supplementary material for: Comparative Transcriptomics of H. pylori Strains AM5, SS1 and Their hpyAVIBM Deletion Mutants: Possible Roles of Cytosine Methylation
Source: PLoS One. 2012 Aug 3;7(8):e42303. doi: 10.1371/journal.pone.0042303 (PMC3411764; doi:10.1371/journal.pone.0042303)
Supplement: Table S1 — Primers used in the study. (DOC) [file pone.0042303.s006.doc]

**Table S1: Primers used in the study**

| S. No. | Primer | Sequence 5´ to 3´ |
| --- | --- | --- |
| 1 | HP0051F1 | GGATCCATGAATTATAAAATTTTA |
| 2 | HP0051R1 | CTCGAGTCATTTTCTTAAGCTTTT |
| 3 | HP0051F2 | GATTTATTTTGTGGGGCTGGG |
| 4 | HP0051R2 | TTGACATGGAGGCCCGCCAA |
| 5 | HP0051F3 | ACATTCTCAATCGCAATAAA |
| 6 | HP0051R3 | ATCTATGCCTTGCCTTTTAGC |
| 7 | Omp11F | ATGATTAAAAGAATTGCTTG |
| 8 | Omp11R | AACATCCAAGTGTTTCCGGC |
| 9 | BabAF | ACCATCAACGAAGCATGCCC |
| 10 | BabAR | TAAAAAGCCTGTAACAAAA |
| 11 | BabBF | GCGTATCAAGCCGTGCTTTT |
| 12 | BabBR | AGGTGTATTTGGTTTGCC |
| 13 | RpoNF | GAAACCTTAGAAGCTCAAAT |
| 14 | RpoNR | TAAAATCTTTAAAGCCTT |
| 15 | FliSF | ATGCAATACGCTAACGCTTA |
| 16 | FliSR | TTAGGCGAGTTCATCTGAATG |
| 17 | FliKF | GCTAGCAACATTCAAGCGGC |
| 18 | FliKR | GATTTTAGGGGCATTTTCTG |
| 19 | VapDF | ATGTATGCTTTAGCGTTTGAT |
| 20 | VapDrR | CTAGGATTTCACAATCTCAG |
| 21 | CagAF | GATTTACTTGATGAAAGGGG |
| 22 | CagAR | GTGAGTTGGTCTTTGTA |
| 23 | VacAF | GTTTGGCGCATTCAAGCAGG |
| 24 | VacAR | CACACGATTATTGATTTCTAA |
| 25 | ArgF | ACGATCACTCAAGAGCGGTG |
| 26 | ArgR | AACCCCAAAGAGCAAAGCTT |
| 27 | FutAF | TTGAGAATGCCTTTGTATTAT |
| 28 | FutAR | TTCAAAACAGAGATTGAACTT |
| 29 | FutBF | TTGAGAATGCCTTTGTATTA |
| 30 | FutBR | TTCAAAACAGAGATTGAACT |
| 31 | FutCF | AAATGCATGGGATTTGATAG |
| 32 | FutCR | GCTCCATGTTTGGCACGCGC |
| 33 | HP16SF | CGGTGGAGCATGTGGTTTAATT |
| 34 | HP16SR | AGGTGTTTTGAAGATTGGCTCC |
| 35 | RAPD1 | ATGACGGAG |
| 36 | RAPD2 | TCGTAGGAG |
| 37 | RAPD3 | GCTTTGGAG |
| 38 | RAPD4 | CACGAGGAG |
